# A2A Receptor Contributes to Tumor Progression in P2X7 Null Mice

**DOI:** 10.3389/fcell.2022.876510

**Published:** 2022-05-18

**Authors:** Elena De Marchi, Anna Pegoraro, Roberta Turiello, Francesco Di Virgilio, Silvana Morello, Elena Adinolfi

**Affiliations:** ^1^ Department of Medical Sciences, University of Ferrara, Ferrara, Italy; ^2^ Department of Pharmacy, University of Salerno, Fisciano, Italy

**Keywords:** P2X7, A2AR, ATP, cancer, immunosuppression, VEGF

## Abstract

ATP and adenosine are key constituents of the tumor niche where they exert opposite and complementary roles. ATP can be released in response to cell damage or actively released by tumor cells and subsequently degraded into adenosine, which accumulates within the tumor microenvironment. Notably, while ATP promotes immune eradicating responses mainly *via* the P2X7 receptor (P2X7R), extracellular adenosine acts as a potent immune suppressor and facilitates neovascularization thanks to the A2A receptor (A2AR). To date, studies exploring the interplay between P2X7R and A2AR in the tumor microenvironment are as yet missing. Here, we show that, in C57/bl6 P2X7 null mice inoculated with B16-F10 melanoma cells, several pro-inflammatory cytokines, including interleukin 1 beta (IL-1β), tumor necrosis factor alpha (TNF-α), interleukin 6 (IL-6), interleukin 12 (IL-12), interleukin 17 (IL-17), interferon gamma (IFN-γ) were significantly decreased, while the immune suppressant transforming growth factor beta (TGF-β) was almost three-fold increased. Interestingly, tumors growing in P2X7-null mice upregulated tumor-associated and splenic A2AR, suggesting that immunosuppression linked to lack of the P2X7R might depend upon A2AR overexpression. Immunohistochemical analysis showed that tumor cells’ A2AR expression was increased, especially around necrotic areas, and that vascular endothelial growth factor (VEGF) and the endothelial marker CD31 were upregulated. A2AR antagonist SCH58261 treatment reduced tumor growth similarly in the P2X7 wild type or null mice strain. However, SCH58261 reduced VEGF only in the P2X7 knock out mice, thus supporting the hypothesis of an A2AR-mediated increase in vascularization observed in the P2X7-null host. SCH58261 administration also significantly reduced intratumor TGF-β levels, thus supporting a key immune suppressive role of A2AR in our model. Altogether, these results indicate that in the absence of host P2X7R, the A2AR favors tumor growth *via* immune suppression and neovascularization. This study shows a novel direct correlation between P2X7R and A2AR in oncogenesis and paves the way for new combined therapies promoting anti-cancer immune responses and reducing tumor vascularization.

## Introduction

The P2X7 receptor (P2X7R) is an ATP gated ion channel known to be central in inflammation for its ability to activate the NLRP3 inflammasome and trigger IL-1β release (Di Virgilio et al., 2017; Orioli et al., 2017; Adinolfi et al., 2018; Pelegrin, 2021). In the context of cancer, P2X7R has been assigned multiple and often contrasting roles as a driver of cancer cell growth (Di Virgilio et al., 2018; Scarpellino et al., 2019) and metastatic dissemination (Di Virgilio et al., 2016; Ulrich et al., 2018; Pegoraro et al., 2021), or as a promoter of immune-mediated tumor eradication (Adinolfi et al., 2015; Adinolfi et al., 2019; De Marchi et al., 2019; Lara et al., 2020). Interestingly, approaches based on either P2X7R antagonism or agonism have proved effective in reducing tumor growth, thus leaving many open questions on the mechanisms underlying P2X7R activity in cancer (Adinolfi et al., 2012; Amoroso et al., 2015; Douguet et al., 2021; Drill et al., 2021). The abundance of P2X7R ligand extracellular ATP (eATP) is a well-recognized characteristic of the tumor microenvironment (TME) (Di Virgilio et al., 2018; De Marchi et al., 2020). The TME is also rich in the ATP hydrolysis derivative extracellular adenosine (eADO) (Boison and Yegutkin, 2019). These two molecules exert opposing actions on the immune system, as, while eATP (*via* P2X7R) is pro-inflammatory and promotes anti-tumor immune response (Kepp et al., 2021), adenosine acts as an immunosuppressant, thus facilitating tumor immune escape (Leone and Emens, 2018; Young et al., 2018; Allard et al., 2020; Antonioli et al., 2021). However, the effects of both eATP and eADO are not limited to activity on immune cells as often through the same receptors expressed by either tumor cells or surrounding stroma they also promote cancer growth, vascularization and metastasis (Di Virgilio et al., 2016; Borea et al., 2018; Scarpellino et al., 2019; Arnaud-Sampaio et al., 2020; Sitkovsky, 2020).

In the TME, eADO accumulation causes suppression of immune effector functions and promotes tumor progression (Allard et al., 2020). These effects are mediated *via* A2A and A2B receptors (A2AR, A2BR), which are expressed by immune stromal and tumor cells. These receptors are both coupled to Gs protein and differ for their ligand binding affinity as the A2AR has a high (Kd: 310 nM), while A2BR has a low affinity (Kd 15 μM) (Yegutkin, 2008). The A2AR, being ubiquitously expressed in the body, especially in the immune system, has emerged as the major adenosine receptor subtype in downregulating inflammation induced by injury, ischemia or infection in a wide variety of diseases (Hasko and Pacher, 2008), in a cyclic AMP (cAMP)/protein kinase A (PKA)-dependent manner, regulating the activity of various transcriptions factors (Nemeth et al., 2003; De Ponti et al., 2007; Ansari et al., 2017; Ahmad et al., 2018). In the context of cancer, the A2AR has been widely studied, emerging as a crucial mediator of eADO effects in the TME (Ohta et al., 2006; Boison and Yegutkin, 2019). Its activation potently suppresses both B and T cells’ effector activity while promoting the accumulation of regulatory T cells (Tregs), which are known to be immunosuppressive (Sorrentino et al., 2013; Antonioli et al., 2021). Furthermore, stimulation of A2AR, on macrophages populating the tumor lesion, promotes the differentiation of M2-like macrophages, which are the major responsible for the accumulation of vascular endothelial growth factor (VEGF) in the TME and the subsequential angiogenesis (Sitkovsky et al., 2014; Allard et al., 2020). Importantly, A2AR not only promotes the establishment of a pro-tumorigenic environment driving macrophage differentiation but also mediates the direct effects of eADO on tumor cells, as shown by the finding that in animal models, tumor cell A2AR promotes invasiveness, motility and proliferation (Beavis et al., 2013; Koszalka et al., 2016; Allard et al., 2019; Kamai et al., 2021).

The eATP and eADO levels are strictly intertwined. In the extracellular milieu, eADO is generated starting from eATP *via* two ectoenzymes, CD39 and CD73, which are also upregulated in the tumor lesion, favoring cancer growth through eADO-mediated immune suppression (Allard et al., 2020; Battastini et al., 2021). We have recently demonstrated that, in an implanted murine melanoma model, P2X7R genetic knockdown or pharmacological antagonism modulates CD39 and CD73 levels on Tregs, CD4^+^ effector lymphocytes, macrophage and dendritic cells (De Marchi et al., 2019). Changes in ectonucleotidases expression correlate with alterations in eATP levels (De Marchi et al., 2019), strongly suggesting that P2X7R expression and function affect the eATP/eADO axis in the TME. Independent studies also indicated a key role of the P2X7R in anti-tumor, CD39-targeting therapies (Li et al., 2019; Yan et al., 2020). Despite this wealth of studies, to our knowledge, no studies demonstrating a correlation between P2X7R and A2AR activity in cancer have been carried out so far. Here we show that the A2AR is upregulated in melanoma bearing P2X7R-null mice and highlight a pivotal role for the A2AR in immune suppression and neovascularization promoted by the absence of the P2X7R. We thus demonstrate, for the first time, a P2X7R-A2AR centered crosstalk in tumorigenesis.

## Materials and Methods

### Cell Cultures

Mouse B16-F10 (ATCC CRL-6475) melanoma cell lines were purchased from Sigma-Aldrich and periodically tested with MycoAlertTM kit (Lonza, Switzerland). B16-F10 were grown in RPMI-1640 medium (Sigma-Aldrich) plus non-essential amino acids (Sigma-Aldrich), FBS (10%), penicillin (100 U/ml), and streptomycin (100 mg/ml) all purchased from Euroclone, Milan, Italy.

### Experiments in Murine Models

*In vivo* experiments were performed with a *p2x7* ^*−/−*^ mice in the C57bl/6 strain and corresponding wild type controls (Adinolfi et al., 2015; De Marchi et al., 2019). Based on calculations performed with the G-power software (Faul et al., 2007) on previously published data (De Marchi et al., 2019), a minimal sample size of six animals per group was chosen to achieve a predicted power of 0.85 with an effect size of 1.78 using a two-tailed t-test. C57bl/6 mice were fed with standard rodent chow diet and water *ad libitum* and were given 1 week to acclimatize after their delivery to the animal facility. The animals were maintained at 22–24°C with a 12 h light cycle in a specific pathogen-free environment. B16-F10 cells (2.5 × 10^5^) were subcutaneously injected into the right flank of each mouse. The animals were weighed and randomised in groups of 3–4 per cage, thanks to the free software available at random.org. Mice present in each cage received the same treatment and the operator was blinded to the group of allocation. Tumors were measured with a calliper, and their volume calculated according to the following equation: volume = π/6 [w1 × (w2)^2^], were w1 = major diameter and w2 = minor diameter. A2AR antagonist SCH58261 (Tocris, bio-techne) or placebo (sterile PBS containing 0.005% DMSO) were intra-peritoneum injected every 3 days after first tumor mass detection (day five from inoculum). The volume of drug administered was based on body weight of each mouse. SCH58261 dose (1 mg/kg) and administration schedule were similar to those previously described in literature (Beavis et al., 2013; Young et al., 2016; Ma et al., 2017). Mice blood samples were collected from the submandibular vein and complemented with 1.5 mg/ml EDTA, immediately before sacrifice (post inoculum day 14). Tumors were post-mortem excised and processed for further analysis. Mice plasma was collected by centrifugation of blood (1,000 × g, 10 min at 4°C). Halt Protease and Phosphatase Inhibitor Cocktail (Thermo Scientific) was added to the samples before storage at −80°C. All animal procedures were approved by the Organism for the animal wellbeing (OPBA) of the University of Ferrara and the Italian Ministry of Health in compliance with EU Directive 2010/63/EU and Italian D.Lgs 26/2014.

### Cytokines and Growth Factors Evaluation

Cytokines levels were evaluated within diluted plasma (1:2) either with mouse interleukin (IL)-1β, tumor necrosis factor (TNF)-α, transforming growth factor (TGF)-β1 (Boster, distributed by Tema Ricerca, Bologna, Italy), interferon (IFN)-γ (R&D, bio-techne) ELISA kits or with Luminex mouse multi-cytokine assay kit (R&D, bio-techne) as per manufacturer’s instructions. Tumor mass were homogenised in lysis buffer (300 μM sucrose, 1 mM K_2_HPO_4_, 1 mM MgSO_4_, 5.5 mM glucose, 20 mM HEPES (pH 7.4), Halt^TM^ Protease and Phosphatase inhibitor cocktail, EDTA-free 100 × (Thermo Fisher), IGEPAL CA-630 0.5%) and loaded for VEGF and TGF-β1 analysis in a 1:5 ratio with mouse ELISA kits (Boster, distributed by Tema Ricerca, Bologna, Italy).

### Immunoblotting

Tumor or organs were homogenised in lysis buffer (300 μM sucrose, 1 mM K_2_HPO_4_, 1 mM MgSO_4_, 5.5 mM glucose, 20 mM HEPES (pH 7.4), Halt^TM^ Protease and Phosphatase inhibitor cocktail, EDTA-free 100 × (Thermo Fisher), IGEPAL CA-630 0.5%). The lysates were loaded in 4–12% NuPAGE Bis-Tris precast gels (Thermo Scientific). Following electrophoretic separation we perfomed transfer blotting on nitrocellulose membranes (Amersham Protran, GE Healthcare, United States); which were subsequently incubated overnight at 4°C with primary antibodies as follows: anti-P2X7 antibody (P8232, Sigma-Aldrich) was diluted 1:300 (1.3 μg/ml), anti-A2AR antibody (sc-32261 Santa Cruz Biotechnology, Heidelberg, Germany) was diluted 1:500 (0.4 μg/ml), anti-myosin II antibody (M8064 Sigma-Aldrich) was diluted 1:1000 (1.5 μg/ml), anti-GAPDH antibody (#6C5-sc32233, Santa Cruz Biotechnology) was diluted 1:1000 (0.1 μg/ml) and anti-β actin antibody (#E-ab-20031, Elabscience) was diluted 1:2500 (0.4 μg/ml). Goat anti-rabbit (170-6515, BIORAD) or goat anti-mouse (170-6516, BIORAD), HRP-conjugated antibodies were applied at a 1:3000 dilution (0.5 μg/ml) as secondary antibodies. Protein bands were visualised by ECL HRP Chemiluminescent Substrate WESTAR RC ULTRA 2.0 or WESTARN NOVA 2.0 (Cyanagen Srl, Bologna, Italy) with a Licor C-Digit Model 3600. Densitometric analysis was carried out with ImageJ software, and data were normalised on the appropriate housekeeping protein (myosin II, GAPDH or β actin).

### Immunohistochemistry

Excised tumors were fixed in Bouin (Sigma-Aldrich) for 7 h at 4°C, dehydrated in cold-graded ethanol series, cleared in xylene, and embedded in paraffin. For immunohistochemistry of A2AR and CD31, serial 7-μm-thick sections were rehydrated, washed in TBS (150 mM NaCl, 50 mM Tris, pH 7.6), incubated for 1 h in blocking solution (TBS 8% FBS, 5% BSA 0.2% TRITON X-100) and subsequently for 16 h at 4°C in blocking solution containing primary antibodies (anti-A2AR mouse antibody, sc-32261 Santa Cruz Biotechnology, at 1:100 (2 μg/ml) and anti-CD31 rat monoclonal antibody, ER-MP12 Thermo Scientific, 1:20 (50 μg/ml)). For detection of A2AR and CD31, heat-induced epitope retrieval was performed using Rodent Decloaker (RD913M Biocare Medical). Slides were then washed twice in TBS plus 0.025% Triton X-100, and endogenous peroxidase activity was blocked by a 10-min incubation at room temperature in PeroxAbolish Solution (PXA969L Biocare Medical). Sections were then incubated for 1 h at room temperature in the blocking solution containing the diluted secondary antibodies [HRP-conjugated goat anti-mouse IgG, P0447, Dako, 1:100 (10 μg/ml), or rabbit anti-rat IgG, P0448, Dako 1:100 (2.5 μg/ml)]. Slides were washed twice in TBS, and peroxidase activity was detected with Liquid DAB Substrate Chromogen System (Dako). Nuclear counterstaining was obtained with Mayer hematoxylin. Following dehydration, sections were mounted with EUKITT (Kindler GmbH), and images acquired thanks to NIS-elements software with a Nikon Eclipse 90i digital microscope (Nikon Instruments, Europe). The percentage of positive cells was quantified using the QuPath free software for immunohistochemical analysis (Bankhead et al., 2017) as previously described (Tattersall et al., 2021).

### Statistics

All data are shown as mean ± standard error of the mean (SEM). Significance was calculated assuming equal standard deviations and variance with a two-tailed Student’s t-test, performed with the GraphPad Prism software V.6.0 (GraphPad, San Diego, Ca, United States). *p*-Values lower than 0.05 were considered statistically significant.

## Results

### P2X7R Deletion Leads to a Decrease in Systemic Levels of Pro-Inflammatory Cytokines and to an Increase of TGF-β

**FIGURE 1:**
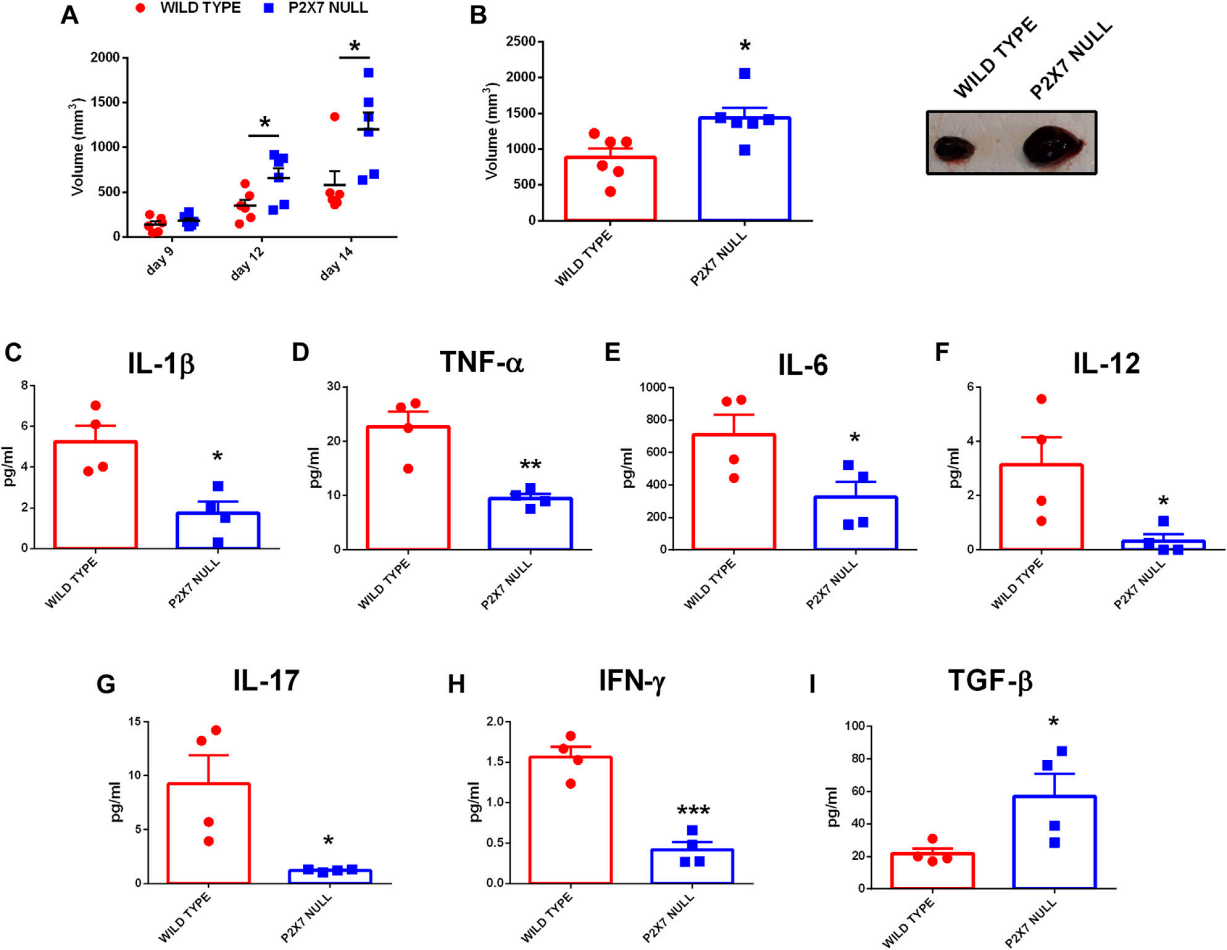
C57bl/6 mice were inoculated into the right flank with B16-F10 cells in wild type and P2X7-null mice. **(A)** Tumor volume was *in vivo* assessed at the indicated time points. **(B)** *Ex-vivo* tumor volume assessed by calliper. **(C)** Representative pictures of tumors from wild type and P2X7 null mice at post-inoculum day 14 (*n* = 6 per group). **(D–J)** Levels of plasma cytokines of tumor-bearing C57bl/6 mice inoculated with B16 cells. IL-1β **(D)**, TNF-α **(E)**, IL-6 **(F)**, IL-12 **(G)**, IL-17 **(H)**, IFN-γ **(I)** and TGF-β **(J)** were evaluated in plasma samples obtained at post-inoculum day 14 (*n* = 4 per group). Data are shown as the mean ± SEM. *p < 0.05, **p < 0.01 and ***p < 0.001.

In recent years we and others have demonstrated that, in mice lacking the P2X7R, solid and liquid tumors show an increased growth due to reduced immune cell infiltration and lower eATP levels (Adinolfi et al., 2015; Hofman et al., 2015; De Marchi et al., 2019). However, an in-depth analysis of the systemic levels of several pro or anti-inflammatory cytokines in tumor-bearing P2X7R null mice has never been carried out. Figure 1 shows that, in parallel with an accelerated tumor growth (Figures 1A–C), P2X7R null mice also show a generalized reduction of systemic pro-inflammatory cytokines (Figures 1D–I) as well as an increase of the immune suppressant TGF-β (Figure 1J). Notably, blood concentration of IL-6 (Figure 1F), IL-12 (Figure 1G), IL-17 (Figure 1H), and IFN-γ (Figure 1I) was strikingly decreased (more than halved), suggesting a substantial impairment of anti-tumoral immune responses. On the contrary, blood TGF-β levels were almost triplicated (Figure 1J). Taken together, these data strongly suggest that the increased tumor progression observed in P2X7R null mice could be linked to a downmodulation of the tumor-eradicating immune response.

### The A2AR is Overexpressed in Tumor-Bearing P2X7R Null Mice

**FIGURE 2:**
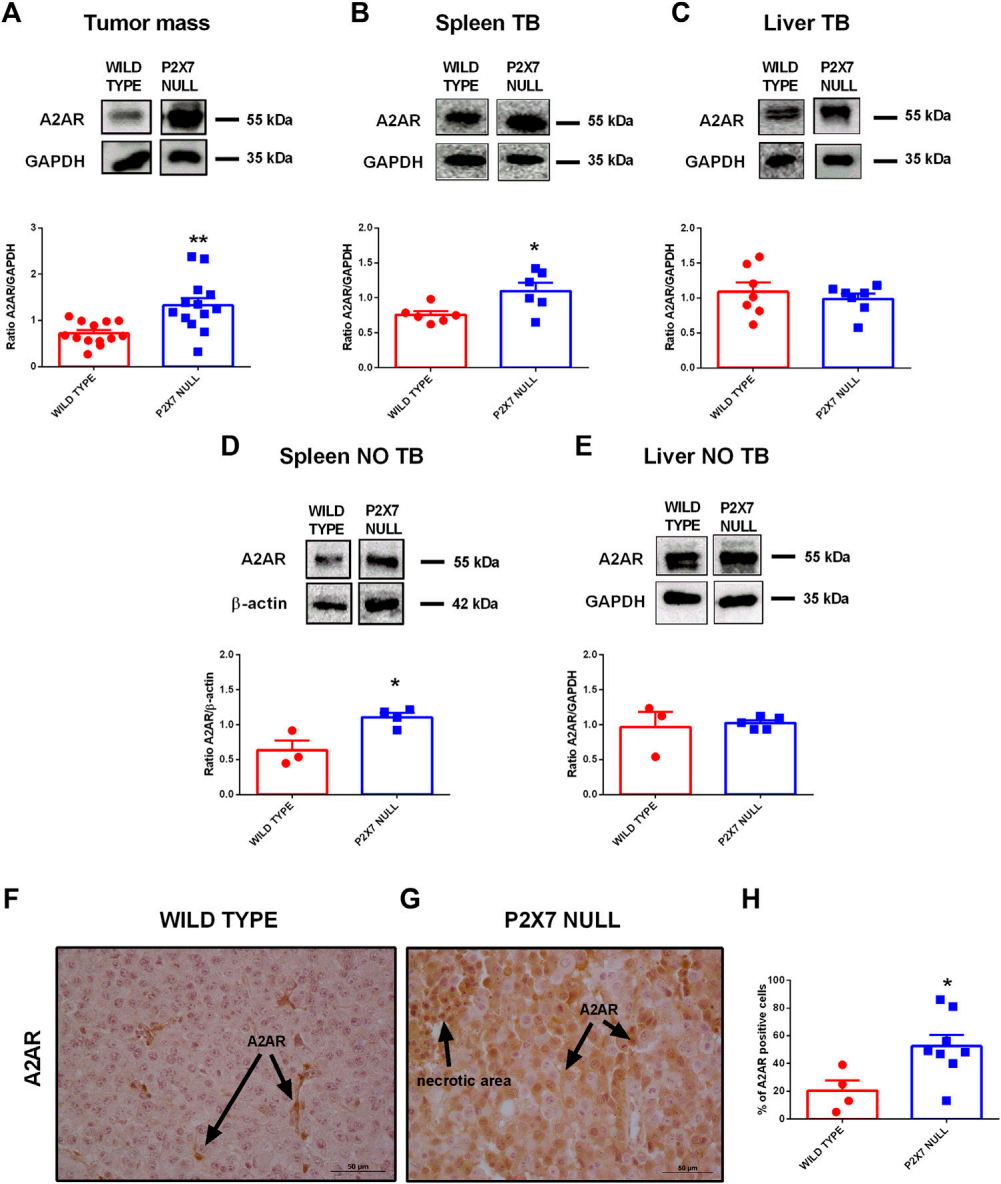
**(A–E)** Western Blot and relative quantification of A2AR in tumor **(A)**, spleen **(B,D)** and liver **(C,E)** derived from wild type and P2X7 null tumor-bearing **(A–C)** or non-tumor-bearing **(D,E)** C57bl/6 mice (*n* = 3–13 per group). **(F–H)** Immunohistochemistry **(F,G)** and relative quantification **(H)** of A2AR in tumor derived from wild type **(F)** and P2X7 null **(G)** C57bl/6 mice (wild type *n* = 4, P2X7 null *n* = 8). Pictures are obtained with a 40X objective. Data are shown as the mean ± SEM.*p < 0.05 and **p < 0.01.

Tumor immune suppression, in analogy to that observed in the P2X7R null melanoma model, has been associated with the accumulation of eADO in the TME and the ensuing activation of the A2AR (Ohta et al., 2006; Ohta and Sitkovsky, 2014). Moreover, decreased eATP concentration accompanied by increased CD73 and CD39 expression strongly support eADO accumulation in the TME of P2X7R null mice. However, P2X7R and A2AR reciprocal crosstalk in the TME was never been investigated before. Therefore, we analyzed A2AR expression levels in tumors growing in either WT or P2X7 null mice observing a significant increase of tumor A2AR expression (Figure 2A). Interestingly, A2AR expression was also higher in the spleens, but not in the livers, from *p2x7* ^*−/−*^ mice whether in tumor-bearing or tumor free than WT mice (Figures 2B–E).

**FIGURE 3:**
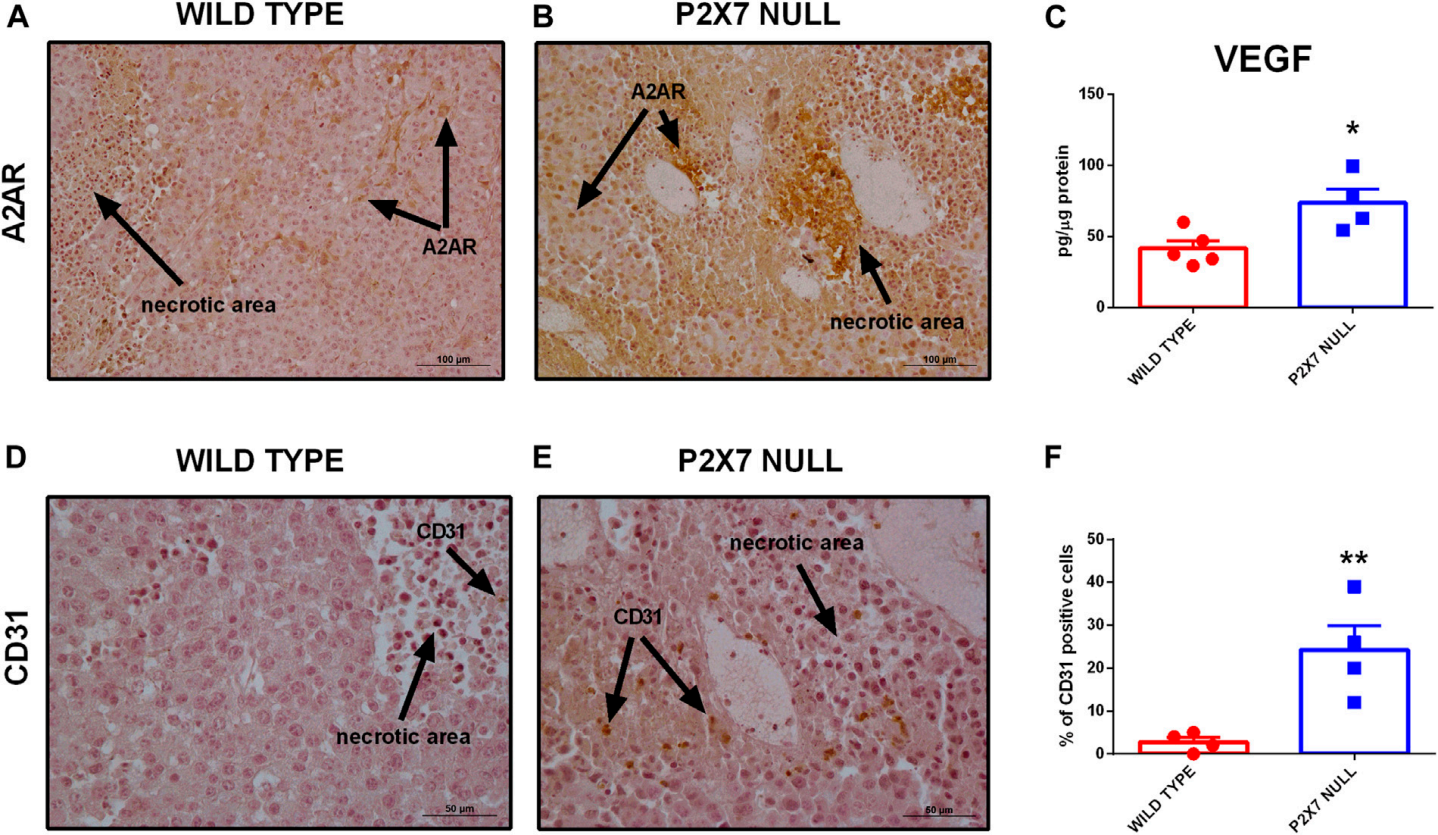
**(A,B)** Immunohistochemistry of A2AR in tumors derived from wild type **(A)** and P2X7 null **(B)** C57bl/6 mice. Pictures are obtained with a 20X objective. **(C)** Intra-tumor levels of VEGF (wild type *n* = 5, P2X7 null *n* = 4). **(D–F)** Immunohistochemistry **(D,E)** and relative quantification **(F)** of CD31 in tumor derived from wild type and P2X7 null C57bl/6 mice (*n* = 4 per group). Data are shown as the mean ± SEM. *p < 0.05 and **p < 0.01.

Immunohistochemical analysis confirmed overexpression of A2AR in tumors from P2X7R-null mice (Figures 2F–H), mainly on tumor cells (Figures 2F,G) and in the periphery of necrotic sites (Figures 3A,B). This observation leds us to investigate tumor levels of the angiogenetic factor VEGF. VEGF is upregulated in hypoxic regions associated with necrosis, and can be secreted in response to A2AR stimulation. Figure 3 shows a significantly higher intra-mass accumulation of VEGF in tumors growing in P2X7R-null mice than WT controls (Figure 3C). The high VEGF tumor levels were paralleled by an augmented expression of the endothelial marker CD31 (Figures 3D–F), indicating increased intratumor blood vessel formation.

### A2AR Blockade Reduces Tumor Growth in P2X7R Genetically Deleted Mice and Normalizes Vascular Endothelial Growth Factor and TGF-β Levels

**FIGURE 4:**
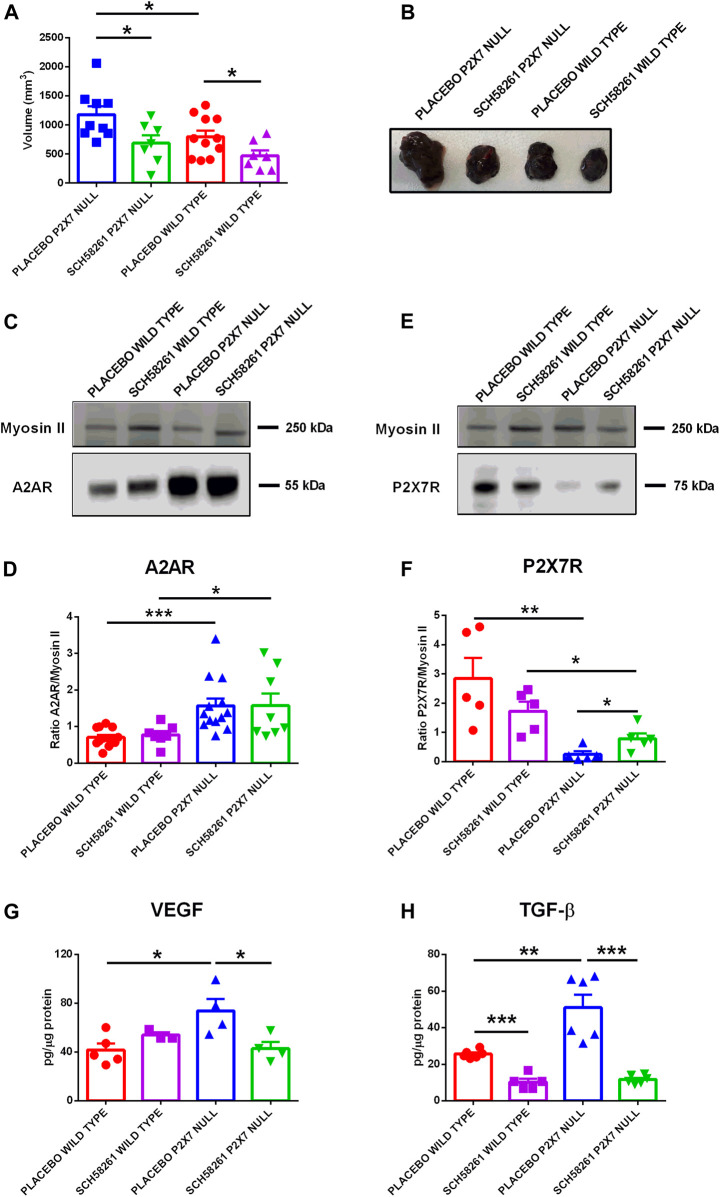
C57bl/6 mice were inoculated into the right flank with B16-F10 cells in wild type and P2X7 null mice. A2AR antagonist SCH58261 (1 mg/kg) or placebo (sterile PBS containing 0.005% DMSO) were intra-peritoneum injected every 3 days after first tumor mass detection (day 5 from inoculum). **(A)** *Ex-vivo* tumor volume assessed by calliper. **(B)** Representative pictures of tumors derived from treated wild type and P2X7 null mice at post inoculum day 14 (*n* = 7–11 per group). **(C–F)** A2AR **(C)** and P2X7R **(E)** expression by Western Blot and relative quantification **(D,F)** (*n* = 5–13 per group). VEGF **(G)** and TGF-β **(H)** were evaluated in tumor masses obtained at post inoculum day 14 (*n* = 3–6 per group). Data are shown as the mean ± SEM. *p < 0.05, **p < 0.01 and ***p < 0.001.

To better understand the role played by A2AR in tumor progression in P2X7R-null mice, we administered the A2AR antagonist SCH58261 to *p2x7* ^*−/−*^ and WT tumor-bearing mice. This drug was administered by intraperitoneal injections at a 1 mg/kg dose every 3 days from the first appearance of the tumor mass. The A2AR antagonist reduced tumor growth in both mice strains (Figures 4A,B), thus demonstrating the central role of A2AR in the B16-F10 melanoma model. Interestingly, SCH58261 treatment did not affect the tumor levels of A2AR, which remained significantly higher in P2X7R-null versus WT mice (Figures 4C,D). On the other hand, the level of P2X7R expressed by tumor cells increased in P2X7 null mice following A2AR antagonism (Figures 4E,F), thus further supporting an interdependency of the two receptors in the TME. It has to be noted that B16-F10 cells injected in P2X7R-null mice still express P2X7R and are, therefore, responsible for the detection of the protein shown in Figures 4E,F. A2AR antagonism also reduced VEGF levels only in P2X7 devoid hosts (Figure 4G). On the other hand, SCH58261 treatment significantly reduced TGF-β levels in tumors growing in both mice strains, further emphasizing the leading role of A2AR in generating an immune-suppressed TME.

## Discussion

The TME is the privileged site of host-tumor interaction and, as such, a key determinant of cancer progression and metastatic spreading. Properties of the TME are also dictated by its features, such as hypoxia that can determine tumor vascularization causing the release of VEGF. Over the last few years, the abundance of eATP was unexpectedly identified as a prominent TME characteristic (Di Virgilio et al., 2018). eATP accumulates into the TME as a consequence of cell death or injury and of non-lytic release from tumor and host cells (Di Virgilio et al., 2018). In the TME, eATP ligates its specific cognate receptors (P2Y and P2X), and it is rapidly metabolised by plasma membrane-expressed or soluble ecto-nucleotidases. The semi-final degradation product of eATP is eADO (the final being inosine), a potent immunosuppressive molecule acting at A2AR (Sitkovsky et al., 2014; Vijayan et al., 2017; Boison and Yegutkin, 2019). Thus, eATP is central in the immunoregulation of the TME because, on the one hand, it is an immunostimulant mediator acting at P2Y and P2X receptors, and on the other hand it supports immunosuppression by generating eADO that activates A2AR. Converging evidence supports the view that the P2X7R is the P2 receptor most heavily involved in host-tumor interaction in the TME (Lara et al., 2020). eATP and the P2X7R are “partners in crime” in the TME as eATP ligates P2X7R and triggers P2X7R-mediated responses, among which release of ATP-itself, controlling the concentration of its own agonist (De Marchi et al., 2019). We recently demonstrated a substantial reduction in eATP levels in tumor-bearing mice devoid of P2X7R. This reduction was also accompanied by a significant increase of ectonucleotidases expression by tumor-infiltrating immune cells, in particular Tregs, thus suggesting an upregulation of eADO levels in the TME of mice lacking P2X7R (De Marchi et al., 2019).

In the current study, we deepened these observations focusing on another key actor of the adenosinergic pathway in cancer: the A2AR (Sitkovsky, 2020). Indeed, A2AR role in causing tumor immune suppression in hypoxic tumors is a consolidated notion that recently lead to several clinical trials exploring the anti-cancer therapeutic efficacy of the administration of A2AR antagonists as a stand-alone treatment or in combination with immune checkpoints inhibitors (Boison and Yegutkin, 2019; Fong et al., 2020; Franco et al., 2021; Augustin et al., 2022). Interestingly, in the TME A2AR activation has been reported to reduce the production of those tumor eradicating cytokines IL-6, IL-17, and IFN-γ that in the present study we found downmodulated in tumor-bearing *p2x7* ^*−/−*^ mice (Augustin et al., 2022). We also observed a reduction of other pro-inflammatory cytokines (IL-1β, TNF-α, and IL-12) related to P2X7R activation and, in general, to innate and type 1 immune response (Correa et al., 2017; Orioli et al., 2017). This immune, cancer-promoting scenario prompted us to further analyse A2AR levels in B16-F10 melanoma-bearing *p2x7* ^−/−^ and WT mice model. Our data show that in P2X7-null mice A2AR expression was increased in the tumor cells and in immunocompetent tissues such as the spleen. However, A2AR expression was also increased in the spleen of non-tumor-bearing P2X7-null mice, strongly suggesting that P2X7R deletion is a prime driver of A2AR upregulation in the immune compartment. In support of this hypothesis, no increased A2AR expression was found in liver and other non-immune tissues from *p2x7* ^*−/−*^ mice (not shown). Of interest, A2AR inhibition was able to increase P2X7R expression by B16-F10 cells, thus suggesting that the compensatory mechanism leading to upregulation of one of the two receptors when the other is downmodulated or blocked might be reciprocal.

A2AR is also known to promote neovascularisation and immune suppression *via* VEGF and TGF-β release (Steingold and Hatfield, 2020) both upregulated in melanoma-bearing *p2x7* ^*−/−*^ mice. Moreover, to further stress the pivotal function of the eADO signalling in the tumor hypoxic response, A2AR expression in the *p2x7* ^*−/−*^ mice was enhanced in peri-necrotic areas, together with an enhanced staining by the endothelial marker CD31. Of note, A2AR inhibition normalised intra-tumor VEGF levels only in P2X7-null mice, thus further supporting the hypothesis that the pro-angiogenic phenotype observed in this model was mainly dependent upon A2AR activity. Finally, A2AR blockade inhibited TGF-β release, thus further reducing immune suppression in the TME.

In conclusion, our data unveil a new A2AR-dependent compensatory mechanism emerging in the absence of host P2X7R. Although the underlying molecular mechanisms warrant further future investigation, the upregulated A2AR in P2X7-null mice promotes tumor growth by favoring immune suppression and neovascularization. Therefore, targeting both receptors has the potential to prove more efficacious than a stand-alone therapeutic strategy. Finally, based on previous studies, a combination of P2X7R and A2AR directed drugs could also prove beneficial in association with traditional chemotherapy (Pegoraro et al., 2020; Sitkovsky, 2020) or irradiation (Huang et al., 2020; Zanoni et al., 2022).

## Data Availability

Complete raw data sets presented in this study will be made available upon reasonable request to the corresponding author.
